# EPIPOX: Immunoinformatic Characterization of the Shared T-Cell Epitome between Variola Virus and Related Pathogenic Orthopoxviruses

**DOI:** 10.1155/2015/738020

**Published:** 2015-10-28

**Authors:** Magdalena Molero-Abraham, John-Paul Glutting, Darren R. Flower, Esther M. Lafuente, Pedro A. Reche

**Affiliations:** ^1^School of Medicine, Unit of Immunology, Complutense University of Madrid, Pza. Ramón y Cajal, s/n, 28040 Madrid, Spain; ^2^School of Life and Health Sciences, University of Aston, Aston Triangle, Birmingham B4 7ET, UK

## Abstract

Concerns that variola viruses might be used as bioweapons have renewed the interest in developing new and safer smallpox vaccines. Variola virus genomes are now widely available, allowing computational characterization of the entire T-cell epitome and the use of such information to develop safe and yet effective vaccines. To this end, we identified 124 proteins shared between various species of pathogenic orthopoxviruses including variola minor and major, monkeypox, cowpox, and vaccinia viruses, and we targeted them for T-cell epitope prediction. We recognized 8,106, and 8,483 unique class I and class II MHC-restricted T-cell epitopes that are shared by all mentioned orthopoxviruses. Subsequently, we developed an immunological resource, EPIPOX, upon the predicted T-cell epitome. EPIPOX is freely available online and it has been designed to facilitate reverse vaccinology. Thus, EPIPOX includes key epitope-focused protein annotations: time point expression, presence of leader and transmembrane signals, and known location on outer membrane structures of the infective viruses. These features can be used to select specific T-cell epitopes suitable for experimental validation restricted by single MHC alleles, as combinations thereof, or by MHC supertypes.

## 1. Introduction

Smallpox was a devastating contagious disease that ravaged humankind for millennia, wiping out entire civilizations [1]. The disease was caused by two types of variola virus (VARV), major and minor, which differed greatly in their average mortality rates: 30% versus 1%, respectively. VARV major was the most prevalent form [2, 3]. Systematic vaccination against smallpox began in the early 19th century but the disease lingered until the World Health Organization (WHO) initiated worldwide vaccination campaigns in 1967. The last case was reported in Somalia in 1977 and in May 1980 the WHO declared that smallpox had been eradicated, ceasing vaccination [1, 2]. Eradication was facilitated because there are no animal reservoirs for the virus, as it only infects humans [4].

VARV belongs to the* Orthopox* genus of the* Poxviridae* family, consisting of large double-stranded DNA viruses that replicate in the cytoplasm of infected cells [5, 6]. Poxviruses are large and complex with ~250 genes and a multistage life cycle, producing different infective forms including intracellular mature virions (IMV) and extracellular enveloped virus (EEV) [5, 6]. Humans can be infected by several poxviruses; the closest to VARV that are also pathogenic to humans are vaccinia (VACV), cowpox (CPXV), and monkeypox (MPXV) viruses [7, 8]. The primary reservoir of MPXV is rodents [9], while CPXV has the broadest animal reservoir range of all poxvirus, including cats, dogs, elephants, and rodents [10]. Historically, VAVC has been considered to emerge after repeated passages from an ancestral CPXV [11]. However, phylogenetic studies question that view and there are some speculations that VACV could be a horsepox virus (HPXV) [12]; yet both, the host and origin of VACV, remain unknown [13]. VACV and CPXV infections in humans are generally mild and self-limiting and can induce cross-protective immunity [14]. The observation that CPXV sufferers did not get smallpox led Edward Jenner in 1798 to introduce a method of vaccination through scarifications with* Variolae Vaccinae*, Latin, for CPXV [15]. Immunization with CPXV was eventually displaced by VACV vaccine, which was used subsequently for global smallpox vaccination [12].

As smallpox was eradicated and vaccination ceased, the global population has become increasingly susceptible to both smallpox and zoonosis by orthopoxviruses [8, 9]. People under 30 have no immunity against these viruses and VACV-induced immunity is waning in those that were vaccinated [16]. Despite recommendations by the WHO, stockpiles of smallpox virus had never been destroyed and there are concerns that unregistered stocks could be used as a weapon of bioterrorism [17]. Several features make smallpox a major terrorist threat. It replicates easily, is aerosolizable, and is highly contagious before, during, and after disease onset. Moreover, smallpox is lethal and disfiguring and has already been used as a biological weapon in North America during the French and Indian Wars [18]. Thus, there is renewed interest in the development of vaccines against smallpox, particularly safer ones, since immunization with VACV can result in serious adverse events and it is considered risky in immunocompromised or immune-suppressed individuals [19].

Immune protection against orthopoxviruses requires both B and T cells [20] but the relevance of T cells is paramount. CD8 T cells are required to eliminate infected cells, while help by CD4 T cells is essential to elicit effective humoral responses [21]. Thus, people with dysfunctional humoral responses (e.g., agammaglobulinemia) can be vaccinated with VACV, while those with loss of T cells cannot as they can suffer severe disease [22]. T-cell immune responses are triggered by the recognition of foreign peptides bound to cell surface-expressed major histocompatibility complex (MHC) molecules, also known as human leukocyte antigens, HLA, in humans. CD4 T cells recognize peptides presented by MHC class II (MHC II) molecules while CD8 T cells recognize peptides presented by MHC class I (MHC I) molecules.

Advances in both immunology and genomic analysis offer new possibilities for eliciting immune protection without the requirement for live-virus vaccination and attendant complications. The identification of HLA class I and class II restricted T-cell epitopes (CD8 and CD4 T-cell epitopes, resp.) from poxviruses may allow us to develop safe and yet immunogenic peptide-based vaccines. Here, we describe the identification of protein antigens that are shared between several pathogenic orthopoxviruses, including VARV, MPXV, CPXV, and VACV, and T-cell epitopes that are identical in all selected proteins. This information was used to create a freely accessible web resource, EPIPOX: URL http://imed.med.ucm.es/epipox/, intended to facilitate the design of epitope-based vaccines against orthopoxviruses.

## 2. Materials and Methods

### 2.1. Orthopoxvirus Sequences and Experimentally Defined T-Cell Epitopes

In this study, we used the entire proteomes of 8 orthopoxviruses: VARV major, strain Bangladesh-1975, GenBank Accession: GB: L22579; VARV major, strain India-1967, GB: NC_00161; Variola major minor, strain Garcia-1966, GB: Y16780; Monkeypox virus, strain Zaire-96-I-16, GB: NC_003310; Cowpox virus strain, strain Brighton Red, GB: AF482758, Vaccinia virus, strain Copenhagen, GB: M35027; Vaccinia virus, strain Tian Tan, GB: AF095689; Vaccinia virus, strain Ankara, GB: U94848. The proteomes were obtained from the various translation features of the relevant GenBank genomic records using BIOPERL [23] (Table 1).

We also used experimentally defined poxvirus-specific HLA I and HLA II-restricted T-cell epitopes that were retrieved from the IEDB [24] and EPIMHC [25] databases. We only considered unique T-cell epitope sequences with a size of 9 amino acids that were reported to be identified in humans infected with orthopoxviruses or who were vaccinated. We provide a list of experimentally defined T-cell epitopes as supplementary material in Additional File S1 in Supplementary Material available online at http://dx.doi.org/10.1155/2015/738020.

### 2.2. Protein Sequence Analyses and Annotations

We took VARV major, strain Bangladesh-1975, as the reference for subsequent sequence analyses. We identified proteins with leader signals using SIGNALP [26] and transmembrane regions using TMHMM [27]. We identified protein orthologs using BLAST [27]. Briefly, we first BLAST the reference proteins against formatted databases of each of the remaining orthopoxvirus proteomes. We performed BLAST searches with default settings and considered only the description of the first hit and the corresponding alignment. Subsequently, we selected those protein searches that gave hits in each of the proteomes with identities greater than 60% and identified the corresponding orthologs. We used BIOPERL to parse BLAST hits [23].

Information on the temporal expression of VACV genes was kindly provided by Dr. Lefkowitz from the Poxvirus Bioinformatics Resource Center [28]. The information consisted on annotations identifying those genes that are expressed early (E), intermediate (I), and late (L) during the life cycle of VACV. This information is provided as supplementary material in Additional File S2. In addition, we identified, from the data provided by Dr. Lefkowitz, gene products associated with the outer membranes of VACV IMV and EEV infective forms, as well as those proteins that are part of the VACV virion or CORE. This information is also included as supplementary material in Additional File S2. Protein annotations obtained for VACV were transferred to protein orthologs.

### 2.3. Prediction of T-Cell Epitopes

We predicted MHC I and MHC II peptide binding to anticipate potential CD8 and CD4 T-cell epitopes, respectively. Specifically, we predicted peptide-MHC binding from VARV Bangladesh proteins that are shared between all selected orthopoxviruses using 32 HLA I- and 33 HLA II-allele specific position-specific scoring matrices (PSSMs) [29–31]. For a given protein, we considered the top 2% and 4% of scoring peptides to constitute HLA I- and HLA II-binding peptides, respectively. We only predicted binding for peptides of nine residues; most HLA I-restricted peptides are 9 residues in length and while HLA II-restricted peptides vary in length (9–22 amino acids) they have a core of 9 residues that anchor the peptide in the binding groove of HLA II molecules [30, 32]. We also used N-gram language models to identify whether peptides can be generated from the source antigen by proteasomal cleavage [33]. This information is only relevant to HLA I-binding peptides, since most peptides presented by MHC I are derived from antigens degraded by the proteasome [34].

### 2.4. Database Building and Web Server Implementation

**Figure 1:**
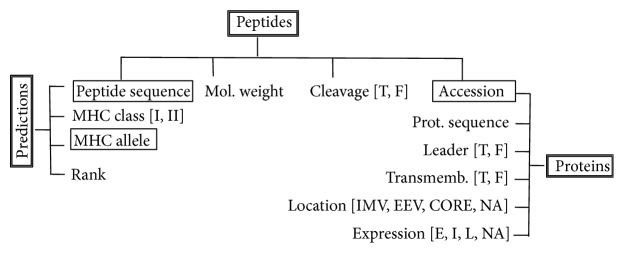
EPIPOX database structure. EPIPOX is a relational database consisting of three main tables:* peptides*,* predictions*, and* proteins*. Table names are boxed with double lines. For each table, we show their fields and boxed with single lines the fields that work as table keys. For fields taking discrete nominal values, we show them between square brackets.

Predicted T-cell epitopes and obtained protein annotations were incorporated into a POSTGRES relational database. The database consists of 3 tables (*peptides*,* predictions*, and* proteins*) that are linked through unique keys (Figure 1). Briefly, table* predictions* contains peptide sequences and their MHC restriction elements; table* peptides* includes the peptide molecular weight, its protein accession number, and whether the peptide is cleaved by the proteasome; and table* proteins* contains gene product information including temporal expression (E: early, I: intermediate, and L:late), location in the virus (IMV, EEV, and CORE), and the existence of leader and transmembrane regions. We also developed a web front end or GUI to allow ready access to EPIPOX. Behind the interface is a Python script that handles database queries through underlying SQL. The EPIPOX resource is implemented on an Apache Web server under the Mac OSX operating system.

## 3. Results and Discussion

### 3.1. Epitope-Vaccine Design against Orthopoxviruses

T-cell adaptive immunity is required for clearance of poxviruses during infection and/or vaccination and can also contribute to protective immunity from subsequent exposures [35, 36]. Moreover, peptides corresponding to VACV-specific CD8 T-cell epitopes can confer protection to mice subjected to lethal VACV challenges [37]. Fueled by the need to develop safer smallpox vaccines, such knowledge has led to the recent identification of many VACV-specific T-cell epitopes [37, 38]. These T-cell epitopes are deposited haphazardly in various specialized databases, including IEDB [24], EPIMHC [25], TEPIDAS [39], and AntiJen [40]. Of relevance for epitope-vaccine design, CD8 T-cells target primarily early and nonstructural gene products [41, 42]. CD4 T cells target late and the most abundant genes products (IMV and EEV membrane proteins and CORE proteins), as do antibodies [42, 43]. While some of the identified VAVC-specific T-cell epitopes are conserved in VARV a rational approach to identifying all potential T-cell epitopes eliciting cross-protective immunity is still required.

### 3.2. Shared Orthopoxvirus Proteins for Cross-Protective Immunity

Nearly all orthopoxviruses can protect against challenge with another orthopoxvirus [14]. This exquisite cross-protective immunity is likely a result of direct antigenic similarity between poxviruses. Therefore, prior to defining potential T-cell epitopes we identified shared antigens between pathogenic orthopoxviruses. Identification of shared antigens is also relevant to reducing the experimental burden associated with T-cell identification. Human pathogen orthopoxviruses have large genomes encompassing over 180 open reading frames (ORF) with the exception of VACV Ankara strain, which has only 157 genes and lacks the ability to replicate [44] (Table 1). Using VARV major, strain Bangladesh-1975, as a reference, we identified 124 ORFs that are shared between 8 different complete genomes from several orthopoxviruses, including VARV minor, CPXV, MPXV, and several VACV strains (Additional File S3). Despite the criterion for selection being 60% identity, all 124 selected proteins have an average identity ≥ 85% as shown in Additional File S3. These proteins are prime candidates to induce cross-protective immunity although they need to be targeted by the immune system. Interestingly, within the selected proteins there are 8 known immunogens that conferred >60% protection to VACV in animal models (Table 2) [45]. Six of these immunogens are IMV or EEV proteins carrying transmembrane regions and/or are being late gene products. Interestingly, among the selected 124 proteins we found 26 additional proteins with transmembrane regions that could also be prime vaccine subunits candidates (Table 3). Some of these proteins also have leader signal sequences (Table 3). Viral proteins with leader sequences follow the cell secretory pathway and are thus also important targets to consider for vaccine design [46, 47].

### 3.3. T-Cell Epitome from Pathogenic Orthopoxvirus Proteins

We targeted the shared orthopoxvirus proteins for T-cell epitopes prediction using 32 and 33 HLA I- and HLA II-specific profile matrices (details in Material and Methods). The alleles targeted for peptide binding prediction are shown in Additional File S4. We selected these alleles because there are experimental peptide-binding data for them, which is required to make accurate peptide-MHC binding predictors [48]. Incidentally, these HLA alleles are frequently expressed in the general population and targeting them for epitope prediction permits the development of epitope-based vaccines covering the entire population. These HLA allelic variants can have overlapping peptide-binding repertoires and can be clustered accordingly in supertypes [49, 50]. Selecting promiscuous peptide-binders to multiple HLA molecules facilitates the development of vaccines with a minimum number of peptides [49–51].

We predicted a total of 18726 HLA I-restricted and 32722 HLA II-restricted orthopoxvirus specific T-cell epitopes, all being identical between all orthopoxviruses considered in this study. In Additional File S4 we provide numbers of T-cell epitopes predicted by each HLA-specific profile used in this study. We predicted more CD4 than CD8 T-cell epitopes because we used a more permissive peptide-binding threshold for MHC II molecules (4% of top scoring peptides) than for MHC I molecules (2% of top scoring peptides) since peptide-binding prediction to MHC II molecules is considerably less accurate than to MHC I molecules [46]. Interestingly, we identified only 8106 unique HLA I-restricted T-cell epitope sequences and a few more (8483) unique HLA II-restricted T-cell epitope sequences. Therefore, there is a considerable overlap between the peptide binding repertoires of HLA molecules, which is larger for HLA II molecules than for HLA I molecules. HLA I-restricted peptides bound on average to 2.3 distinct HLA I molecules, while HLA II-restricted peptides bound on average to 3.8 distinct HLA II molecules. This is due to the fact that peptide-binding to MHC II molecules is more degenerate than to MHC I molecules [29, 30]. In Additional File S5, we provide all distinct predicted peptides with the HLA molecules that they were predicted to bind. Interestingly, there is also some overlap between HLA I- and HLA II-restricted peptides. In particular, we find that there are 2452 peptides that are predicted to be restricted by both HLA I and HLA II molecules. Thus, in total the predicted T-cell epitome consisted of just 14137 unique sequences among all predicted T-cell epitopes.

We compared the predicted T-cell epitome with experimentally defined poxvirus-specific HLA-restricted T-cell epitopes deposited in the IEDB [24] and EPIMHC [25]. We retrieved 170 HLA I and 9 HLA II-restricted T-cell epitopes meeting our criteria (see Additional File S1) but we only considered for comparison 85 HLA I- and 8 HLA II-restricted T-cell epitopes that we identified here to be conserved in all orthopoxviruses considered in this study. Of those, 72 HLA I- and 6 HLA II-restricted T-cell epitopes were found within our predicted T-cell epitome. Moreover, we predicted the experimentally verified restriction element in > 80%. The experimentally determined and shared epitopes that were not predicted (a minority) either are restricted by noncovered alleles or were simply not predicted. In Table 4, we summarize the data showing the verified and predicted HLA restriction elements. In sum, we readily predicted most of the experimentally verified T-cell epitopes. Considering that on average 10% of predicted T-cell epitopes can be experimentally verified [52], we shall expect that there are many more valid T-cell epitopes remaining to be validated within the T-cell epitome predicted in this study.

### 3.4. EPIPOX Database and Web Server

We developed a relational database based upon the predicted T-cell epitome and a web-based resource to facilitate online access and to query the database. We named this resource EPIPOX and made it available for free public use (URL: http://imed.med.ucm.es/epipox/). EPIPOX is a* de facto* analysis pipeline of viral T-cell epitomes. The content of the EPIPOX database is organized in three tables (*peptides*,* predictions*, and* proteins*) (Figure 1). The table* predictions* contains all predicted T-cell epitopes, consisting of 18726 HLA I- and 32722 HLA II-restricted peptides, each identified by its sequence and restriction element. Peptide sequences in this table are not unique as each peptide can bind to numerous HLA I molecules. Peptide sequences are, however, unique in the table* peptides. *This table contains 14137 sequences comprising the whole predicted epitome regardless of the restriction elements. Antigen annotations in EPIPOX are found within the table* proteins* (Figure 1). We only included annotations that are relevant to epitope vaccine design, such as temporal expression of gene products and location in relevant structures of the virus such as the EEV and IVM membranes and CORE. Early expressed proteins and highly expressed proteins are generally thought to be more immunogenic, particularly with regard to CD8 T cells [53, 54]. On the other hand, highly abundant late proteins that are located in membrane structures of the poxvirus appear to be the main focus of the antibody and CD4 T-cell response [42, 43]. In the table* proteins*, we also provide annotations on whether the proteins have transmembrane region or leader signal sequence, as proteins with these features often interact with host cells and are important targets for subunit vaccine design [46, 47].

**Figure 2:**
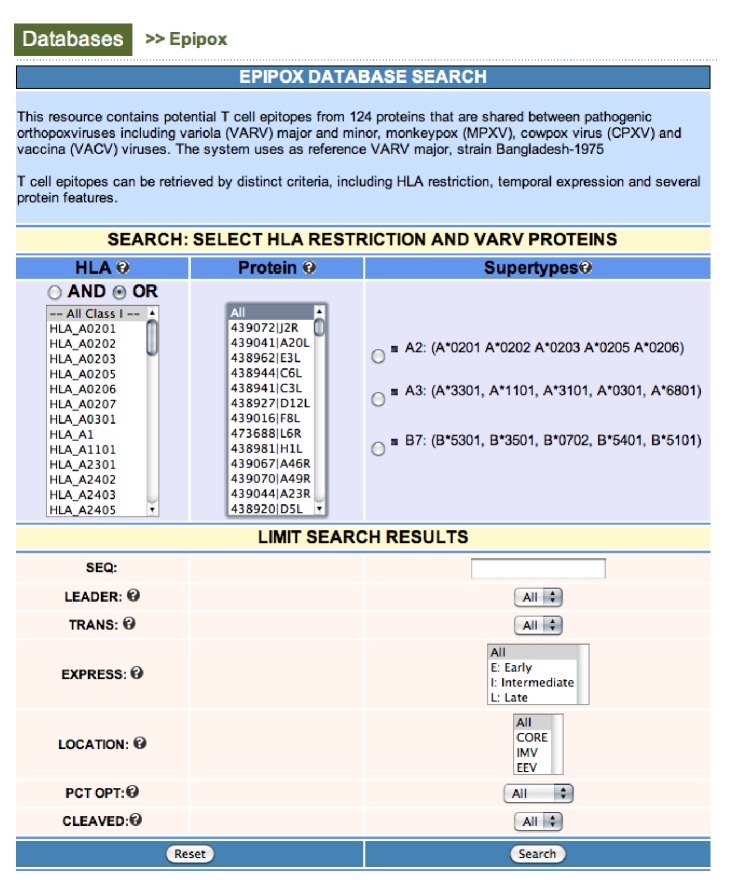
EPIPOX input page. The input page of EPIPOX is divided in two main sections for intuitive use. In the first part (SEARCH), users select HLA molecules and proteins to retrieve T-cell epitopes (multiple selection is allowed) while in the second part the user can limit the search output according to various criteria. These criteria include temporal expression of gene products (E: early; I: intermediate; L: late), location of proteins in relevant structures of the virus (CORE, IMV, and EEV), and the presence of leader and transmembrane regions. In addition, users can select only those peptides with a relative score above some selectable value. HLA-specific profiles used to score T-cell epitopes can reach a maximum score, which is used to set the relative score in percentage of each peptide. For HLA I-restricted epitopes, users can also restrict the search to those epitopes potentially generated by the proteasome.

**Figure 3:**
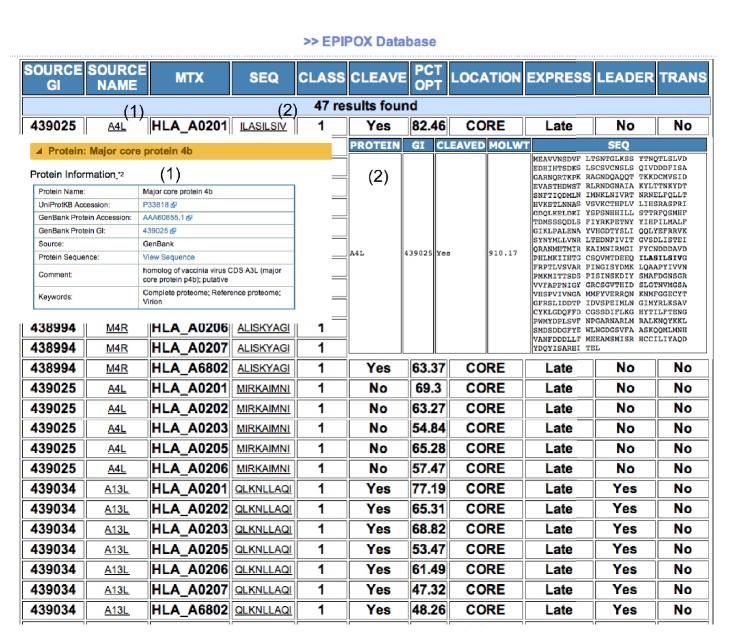
EPIPOX result page. The figure shows a slice of the output resulting from promiscuous CORE protein peptides binding to the A2 supertype. The output consists of a tabulated list, with information on each of the fields of the search query (columns). From field* SOURCE NAME* (1), users can access proteins from the Virus Pathogen Database (http://www.viprbrc.org/) (1) and by clicking on the epitope sequence, field* SEQ* (2), users will get the amino acid sequence of the protein showing the peptide in bold (2).

The EPIPOX web interface (Figure 2) allows querying of the database combining any annotation field in the database, as described above. For intuitive use, the interface is divided in two main sections. In the first section (SEARCH), users select proteins and restriction elements for epitope retrieval. In this section, EPIPOX also provides the option to query the database for promiscuous T-cell epitopes binding to three HLA I supertypes (A2, A3, and B7). The alleles belonging to these supertypes are present in 88% of the population regardless of their ethnic groups. Selecting promiscuous peptides restricted by these 3 supertypes facilitates maximizing the population coverage of vaccines with minimum numbers of peptides [49, 50, 55]. In the second section (LIMIT), users can select annotation criteria to restrict the results. As an example, in Figure 3, we show the page resulting from a sample query consisting of promiscuous peptides from CORE binding to the A2 supertype. From the EPIPOX output, users can also access additional information available from the Virus Pathogen Resource database (Figure 3) [56].

EPIPOX is related somewhat to certain existing databases. On the one hand, it shares features with generic epitope databases such as EPIMHC [25], AntiJen [40], and IEDB [24] and on the other hand it shares features with poxvirus genome annotation-orientated databases such as the *Poxviridae* database [28] (no longer operating) and the Virus Pathogen Resource (http://www.viprbrc.org/) [56]. This later resource contains information on virus sequences, functional annotations and epitopes derived from IEDB [24]. However, the Virus Pathogen Resource does not allow selection of epitopes or antigens by criteria that are relevant to epitope vaccine design. In fact, EPIPOX is the only dedicated immunologic resource that has been designed to facilitate the rational selection of epitopes and antigens for subunit vaccine design.

## 4. Conclusions and Future Development

The availability of the VARV genomes enables the use of predictive tools that reveal entire T-cell epitomes and facilitate the development of epitope-based vaccines. However, in large and complex viruses, such as VARV, the potential T-cell epitome can be so sizeable that it will challenge experimental validation. Therefore, in this work we applied a rational strategy to limit the list of potential T-cell epitopes. First, we reduced the number of antigens by half by simply selecting those that are conserved among pathogenic orthopoxviruses related to VARV. Second, we enriched the antigens with annotations such as temporal expression and location. Lastly, we created a resource and* de facto* analysis pipeline (EPIPOX) with which to interrogate the resulting T-cell epitome and enable users to select immunologically relevant subsets of T-cell epitopes suitable for experimental validation.

We expect that this work and EPIPOX will be instrumental in developing safer smallpox vaccines and thereby in preventing zoonosis caused by other orthopoxviruses, including MPXV, which is also a potential terrorist bioweapon. In the future, we plan to enhance EPIPOX with validated and/or experimentally determined epitopes, upgrade protein annotations with functional information, and include additional features such as TAP transport [57], ERAAP cleavage [58], and T-cell epitope immunodominance. In sum, we would expect EPIPOX to establish itself as a facilitating resource of true utility in* inter alia* immunoinformatic characterization of viral genomics and computational reverse vaccinology.

## Supplementary Material

The supplementary materials provide relevant data collected and resulting from this work. Additional File S1 provides a list of experimentally determined poxvirus-specific T cell epitopes collected from relevant databases. For each epitope sequence, we show the known MHC restriction elements, protein GIs and relevant literature references. Additional File 2 reports known temporal expression (E: early, I: intermediate and L:late) and location of Vaccinia virus proteins. The information was kindly provided by Dr. Elliot J Lefkowitz. Additonal File S3 shows the 124 proteins from Variola major virus, strain Bangladesh, (VARVMJ BSH), that we found to be shared by all pathogenic orthopoxviruses, indicating their counterparts and sequence identity between them. Additional File S4 identifies the HLA I- and HLA II-molecules that were targeted for epitope prediction as well as the number of peptides predicted to bind to each one of them. Finally, Additional File S5 lists all HLA Iand HLA II-restricted T-cell epitopes shared between pathogenic orthopoxviruses predicted in this study. The epitopes are ordered by the number of different HLA molecules that can present them.

## Figures and Tables

**Table 1:** Orthopoxviruses used in this study.

Virus	Strain	ACC	Genes
VARV major	Bangladesh-1975	L22579	189
VARV major	India-1967	NC_00161	197
VARV minor	Garcia-1966	Y16780	206
MPXV	Zaire-96-I-16	NC_003310	191
CPXV	Brighton Red	AF482758	218
VACV	Copenhagen	M35027	262
VACV	Tian Tan	AF095689	243
VACV	Ankara^*∗*^	U94848	157

^*∗*^Modified strain that has lost the ability to replicate; VARV: variola virus; MPXV: monkeypox virus; CPXV: cowpox virus; VACV: vaccinia virus.

**Table 2:** Orthopoxvirus proteins contributing to cross-protective immunity.

VACC: GI|ORF	VARV: GI|ORF	MPXV: GI|ORF	CPXV: GI|ORF	LOC^1^/EXP^2^/TM^3^/LD^4^
335424|L1R	438991|M1R	17974993|M1R	20153082|V099	IMV/late/yes/no
335455|D8L	439016|F8L	17975018|E8L	20153106|V119	IMV/late/yes/no
335500|A27L	439052|A31L	17975052|A29L	20153143|V156	IMV/late/no/no
335508|A33R	439057|A36R	17975058|A35R	20153149|V162	EEV/early/yes/no
335549|B5R	439084|B6R	17975080|B6R	20153177|V190	EEV/#/yes/yes
335438|H3L	439004|I3L	17975006|H3L	20153094|V107	IMV/late/yes/no
335477|A10L	439032|A11L	17975034|A11L	20153122|V135	CORE/#/no/no
335341|C7L	438926|D11L	17974926|D10L	20153015|V028	U/early/no/no

Table shows GenBank identification numbers (GI) and open reading frame names (ORF) for VACC (strain Copenhagen), VARV (strain Bangladesh-1975), MPXV (strain Zaire-96-I-16), and CPXV (strain Brighton Red). ^1^LOC: location, ^2^EXP: temporal expression, ^3^TM: transmembrane, and ^4^LD: leader signal. NS: nonstructural gene. #: information not available. U: unknown. List of proteins was obtained from [45]. Annotations 1, 2, 3, and 4 obtained as indicated elsewhere in Section 2.

**Table 3:** Shared orthopoxvirus proteins with transmembrane and/or leader sequences.

VARV GI|ORF	MPXV GI|ORF	CPXV GI|ORF	VACV GI|ORF	IDEN^1^ (%)	TM^2^	LEAD^3^	EXP^4^	LOCATION^5^
GI:439084|B6R	GI:17975080|B6R	GI:20153177|V190	GI:335549|B5R	93.1	Yes	Yes	L	EEV membrane^*∗*^
GI:439016|F8L	GI:17975018|E8L	GI:20153106|V119	GI:335455|D8L	94.7	Yes	No	L	IMV membrane^*∗*^
GI:438990|H9R	GI:17974992|G10R	GI:20153081|V094	GI:335423|G9R	98.1	Yes	No	L	U
GI:438919|D4R	GI:17974919|D3R	GI:20153007|V020	GI:335333|C11R	88.8	Yes	Yes	U	U
GI:439085|B7R	GI:17975081|B7R	GI:20153178|V191	GI:335550|B6R	93.1	Yes	No	U	U
GI:439035|A14L	GI:17975037|A14L	GI:20153125|V138	GI:335483|A13L	88.6	Yes	No	L	IMV membrane
GI:438946|C8L	GI:17974949|C10L	GI:20153036|V049	GI:335366|F4L	97.6	Yes	No	E	U
GI:438977|K5L	GI:17974979|I5L	GI:20153068|V081	GI:335409|I5L	94.9	Yes	No	L	IMV membrane
GI:438967|E8R	GI:17974969|F7R	GI:20153058|V071	GI:335395|E8R	97.4	Yes	No	L	U
GI:439004|I3L	GI:17975006|H3L	GI:20153094|V107	GI:335438|H3L	95.8	Yes	No	L	IMV membrane^*∗*^
GI:439014|F6R	GI:17975016|E6R	GI:20153104|V117	GI:335453|D6R	99.0	Yes	No	L	U
GI:439003|I2R	GI:17975005|H2R	GI:20153093|V106	GI:335437|H2R	99.2	Yes	No	L	U
GI:439056|A35L	GI:17975057|A34L	GI:20153148|V161	GI:335506|A32L	98.1	Yes	No	L	U
GI:439000|L5L	GI:17975002|L5L	GI:20153090|V103	GI:335433|J5L	98.1	Yes	No	L	U
GI:439058|A37R	GI:17975059|A36R	GI:20153150|V163	GI:335509|A34R	98.1	Yes	No	L	EEV membrane
GI:438991|M1R	GI:17974993|M1R	GI:20153082|V095	GI:335424|L1R	99.2	Yes	No	L	IMV membrane^*∗*^
GI:439057|A36R	GI:17975058|A35R	GI:20153149|V162	GI:335508|A33R	93.0	Yes	No	E	EEV membrane^*∗*^
GI:438951|C13L	GI:17974954|C15L	GI:20153041|V054	GI:335373|F9L	97.5	Yes	No	L	U
GI:439038|A17L	GI:17975040|A17L	GI:20153129|V142	GI:335486|A16L	97.0	Yes	No	L	U
GI:438974|K2L	GI:17974976|I2L	GI:20153065|V078	GI:335405|I2L	99.3	Yes	No	L	U
GI:439008|I7R	GI:17975010|H7R	GI:20153098|V111	GI:335442|H7R	95.2	Yes	No	L	U
GI:438982|H3L	GI:17974984|G2L	GI:20153073|V086	GI:335414|G3L	95.8	Yes	No	L	U
GI:439042|A22L	GI:17975044|A21L	GI:20153134|V147	GI:335490|A21L	96.9	Yes	No	U	U
GI:439059|A38R	GI:17975061|A38R	GI:20153152|V165	GI:335512|A36R	92.3	Yes	No	E, L	EEV membrane
GI:439031|A10L	GI:17975033|A10L	GI:20153121|V134	GI:335476|A9L	89.0	Yes	Yes	E, L	U
GI:439033|A12R	GI:17975035|A12R	GI:20153123|V136	GI:335481|A11R	98.5	Yes	No	L	U
GI:439036|A15L	GI:17975038|A15L	GI:20153126|V139	GI:335484|A14L	97.8	Yes	No	L	IMV membrane
GI:439067|A46R	GI:17975066|A43R	GI:20153159|V172	GI:335522|A43R	92.3	Yes	Yes	U	U
GI:439039|A18L	GI:17975041|A18L	GI:20153130|V143	GI:335487|A17L	98.0	Yes	No	L	IMV membrane
GI:439077|J7R	GI:17975076|B2R	GI:20153172|V185	GI:335539|A56R	82.1	Yes	Yes	E, L	EEV membrane
GI:439062|A41L	GI:17975063|A40L	GI:20153155|V168	GI:335516|A38L	94.7	Yes	Yes	U	U

Table shows GenBank identification numbers (GI) and open reading frame names (ORF) for VARV: strain Bangladesh-1975, MPXV: strain Zaire-96-I-16, CPXV: strain Brighton Red, and VACV: strain Copenhagen. ^1^IDEN: average identity between the selected proteins. ^2^TM: transmembrane. ^3^LEAD: leader signal. ^4^EXP: temporal expression (E: early, I: intermediate, and L: late). ^5^LOCATION: location. ^*∗*^Proteins known to induce protective immunity (see Table 2). Annotations were obtained as indicated elsewhere in Section 2. U: information not found.

**(a):** 

CD8 T-cell epitopes	VARV GI	VARV ORF	Experimental HLA I restriction	Predicted HLA I restriction					
A LMRRIAVV	439013	F5R	HLA-A2	HLA-A0201	HLA-A0202	HLA-A0203	HLA-A0205	HLA-A0206	HLA-A6802
YLLSLFSTL	439056	A35L	HLA-A2	HLA-A0201	HLA-A0202	HLA-A0203	HLA-A0205	HLA-A0206	HLA-A6802
YLAKLTALV	438985	H5R	HLA-A2	HLA-A0201	HLA-A0202	HLA-A0203	HLA-A0205	HLA-A0206	HLA-A6802
NLLCHIYSL	438979	K7L	HLA-A2	HLA-A0201	HLA-A0202	HLA-A0203	HLA-A0205	HLA-A0206	HLA-Cw0702
IVIEAIHTV	439072	J2R	HLA-A0201	HLA-A0201	HLA-A0202	HLA-A0203	HLA-A0205	HLA-A0206	HLA-Cw0304
SLSAYIIRV	439004	I3L	HLA-A0201	HLA-A0201	HLA-A0202	HLA-A0203	HLA-A0205	HLA-A0206	HLA-A0207
YLDGQLARL	438965	E6R	HLA-A0201	HLA-A0201	HLA-A0202	HLA-A0203	HLA-A0205	HLA-A0206	HLA-A0207
YLPEVISTI	438988	H7L	HLA-A0201	HLA-A0201	HLA-A0202	HLA-A0203	HLA-A0205	HLA-A0206	HLA-Cw0102
TYNDHIVNL	439072	J2R	HLA-A2301	HLA-A2301	HLA-A2402	HLA-A2403	HLA-A2405	HLA-A2407	HLA-Cw0702
RPPSFYKPL	439046	A25R	HLA-B7	HLA-B0702	HLA-B3501	HLA-B5101	HLA-B5301	HLA-B5401	HLA-Cw0102
ALDEKLFLI	439045	A24R	HLA-A0201	HLA-A0201	HLA-A0202	HLA-A0203	HLA-A0205	HLA-A0206	HLA-A0207
FPYEGGKVF	438968	E9L	HLA-B0702	HLA-B0702	HLA-B1502	HLA-B3501	HLA-B5101	HLA-B5301	HLA-B5401
RLYDYFTRV	438973	K1L	HLA-A0201, HLA-A2	HLA-A0201	HLA-A0202	HLA-A0203	HLA-A0205	HLA-A0206	
ILDDNLYKV	438985	H5R	HLA-A0201, HLA-A2	HLA-A0201	HLA-A0202	HLA-A0205	HLA-A0207	HLA-Cw0702	
LLSYYVVYV	439009	F1R	HLA-A2	HLA-A0201	HLA-A0202	HLA-A0203	HLA-A0205	HLA-A0206	
FLIDLAFLI	438960	E1L	HLA-A2	HLA-A0202	HLA-A0203	HLA-A0205	HLA-A0206	HLA-Cw0304	
FPRSMLSIF	438994	M4R	HLA-B07:02	HLA-B0702	HLA-B3501	HLA-B4402	HLA-B5301	HLA-B5401	
YLFDFVISL	438996	L1R	HLA-A2	HLA-A0201	HLA-A0202	HLA-A0203	HLA-A0205	HLA-A0206	
YLIKLIEPV	439009	F1R	HLA-A0201, HLA-A2	HLA-A0201	HLA-A0202	HLA-A0203	HLA-A0205	HLA-A0206	
SPSNHHILL	439025	A4L	HLA-B07:02	HLA-B0702	HLA-B3501	HLA-B5101	HLA-B5301	HLA-B5401	
YPSNKNYEI	439033	A12R	HLA-B07:02	HLA-B0702	HLA-B3501	HLA-B5101	HLA-B5301	HLA-B5401	
MLMETMFFI	439007	I6R	HLA-A2	HLA-A0201	HLA-A0202	HLA-A0203	HLA-A0205	HLA-A0206	
ILNPVASSL	438998	L3R	HLA-A2	HLA-A0201	HLA-A0202	HLA-A0205	HLA-A0206	HLA-B1501	
FPSVFINPI	438968	E9L	HLA-B0702	HLA-B0702	HLA-B3501	HLA-B5101	HLA-B5301	HLA-B5401	
KYQSPVNIF	439043	A21R	HLA-A24, HLA-class I	HLA-A2301	HLA-A2402	HLA-A2403	HLA-A2405	HLA-A2407	
YLFGGFSTL	438980	K8R	HLA-A2	HLA-A0201	HLA-A0202	HLA-A0203	HLA-A0205	HLA-A0206	
YLYETYHLI	438981	H1L	HLA-A2	HLA-A0201	HLA-A0202	HLA-A0205	HLA-A0206		
VLYNGVNYL	439009	F1R	HLA-A2	HLA-A0201	HLA-A0202	HLA-A0205	HLA-A0206		
LIQEIVHEV	439029	A8L	HLA-A0201	HLA-A0201	HLA-A0202	HLA-A0205	HLA-A0206		
VELGSGNSF	439043	A21R	HLA-B3701	HLA-B1501	HLA-B1502	HLA-B4402	HLA-Cw0304		
RMIAISAKV	438934	P1L	HLA-A2	HLA-A0201	HLA-A0202	HLA-A0203	HLA-A0206		
FILGIIITV	439036	A15L	HLA-A0201	HLA-A0202	HLA-A0203	HLA-A0205	HLA-A0206		
LLSKNTFYL	438981	H1L	HLA-A2	HLA-A0201	HLA-A0202	HLA-A0205	HLA-A0207		
RPRDAIRFL	438961	E2L	HLA-B0702	HLA-B0702	HLA-B3501	HLA-B5301	HLA-B5401		
KLFNKVPIV	438996	L1R	HLA-A2	HLA-A0201	HLA-A0202	HLA-A0206	HLA-A6802		
SLFMILCTR	438979	K7L	HLA-A0301, HLA-A1101	HLA-A1101	HLA-A3101	HLA-A3301	HLA-A6801		
ILNDEQLNL	439029	A8L	HLA-A0201	HLA-A0201	HLA-A0205	HLA-A0207			
YLLGDSDSV	439038	A17L	HLA-A2	HLA-A0201	HLA-A0205	HLA-A0206			
QLMYALEPR	438985	H5R	HLA-A0301, HLA-A1101	HLA-A3101	HLA-A3301	HLA-A6801			
LMDENTYAM	439066	A45R	HLA-A2	HLA-A0205	HLA-A0207	HLA-Cw0702			
GLLLGCFWV	438955	C17L	HLA-A2	HLA-A0202	HLA-A0203	HLA-A0206			
LLSHFYPAV	438952	C14L	HLA-A2	HLA-A0201	HLA-A0205	HLA-A6802			
NLFTFLHEI	439045	A24R	HLA-class I	HLA-A0201	HLA-A0203	HLA-A0206			
GLFDFVNFV	439070	A49R	HLA-A2, HLA-A0201	HLA-A0202	HLA-A0203	HLA-A0206			
YLGPRVCWL	439038	A17L	HLA-A2	HLA-A0202	HLA-A0205	HLA-A0206			
ILKSLGFKV	438980	K8R	HLA-A2	HLA-A0202	HLA-A0203	HLA-A0205			
DEVASTHDW	439025	A4L	HLA-B4403	HLA-B4402	HLA-B5801				
FLVIAINAM	439029	A8L	HLA-A2, HLA-A0201	HLA-A0206	HLA-Cw0304				
LSDLKKTIY	439040	A19R	HLA-A1, HLA-class I	HLA-A0101	HLA-B1501				
LLYFKVFGI	439019	N1L	HLA-A2	HLA-A0202	HLA-A0205				
SSNIMSESY	439014	F6R	HLA-A0101, HLA-A3002	HLA-A0101	HLA-B1501				
DTRGIFSAY	439032	A11L	HLA-A26, HLA-class I	HLA-A0101	HLA-B5801				
QIDVEKKIV	439029	A8L	HLA-A0201	HLA-A0207	HLA-A6802				
YLFRCVDAV	439030	A9R	HLA-A2	HLA-A0205	HLA-A0206				
KIEDLINQL	438973	K1L	HLA-A0201	HLA-A0203	HLA-A0207				
FTIDFKLKY	439009	F1R	HLA-A1, HLA-A2601, HLA-A2902	HLA-A0101	HLA-Cw0702				
WLKIKRDYL	439074	J4R	HLA-B0801	HLA-B0801					
AINVEKIEL	473688	L6R	HLA-A0201	HLA-A0207					
RIFVRVYNV	439007	I6R	HLA-A2	HLA-A0202					
SIIDLIDEY	438942	C4R	HLA-B1501	HLA-A0203					
EERHIFLDY	439009	F1R	HLA-B4403	HLA-B4402					
ILSDENYLL	439028	A7L	HLA-A2, HLA-A0201	HLA-A0205					
HISALKRRY	439027	A6R	HLA-A0101, HLA-A2902	HLA-A0101					
FLNISWFYI	438968	E9L	HLA-A2, HLA-A02:01	HLA-A0205					
SEVKFKYVL	439002	I1L	HLA-B44	HLA-B4402					
KLLLWFNYL	438980	K8R	HLA-A2	HLA-A0203					
YIDISDVKV	438973	K1L	HLA-A0201	HLA-A0207					
VWINNSWKF	439013	F5R	HLA-A24, HLA-A2301, HLA-A2402	HLA-Cw0702					
VLPFDIKKL	439014	F6R	HLA-A0201	HLA-Cw0102					
VETSISDYY	439043	A21R	HLA-B3701	HLA-B1501					
SQIIDISLR	439022	A1L	HLA-A0301, HLA-A1101	HLA-B2705					
HDVYGVSNF	439045	A24R	HLA-B4403	HLA-B4402					

**(b):** 

CD4 T-cell epitope	VARV GI	VARV ORF	Experimental HLA II restriction	Predicted HLA II restriction									
FLIDLAFLI	438960	E1L	HLA-class II	DRB1*∗*0101	DRB1*∗*0102	DRB1*∗*0301	DRB1*∗*0311	DRB1*∗*0401	DRB1*∗*1201	DRB1*∗*1302	DRB1*∗*1502	DRB3*∗*0101	DRB5*∗*0101
IHWQIISSE	439072	J2R	DRB1*∗*04:05	DRB1*∗*0405	DRB1*∗*0406	DRB1*∗*0410	DRB1*∗*1304	DRB3*∗*0101					
YIDAYVSRL	439021	N3L	DRB1*∗*15:01	DRB1*∗*0901	DRB1*∗*1103	DRB3*∗*0202							
LMDENTYAM	439066	A45R	HLA-class II	DRB1*∗*1103									
IDAYVSRLL	439021	N3L	DRB1*∗*15:01	DRB1*∗*1201									
RMIAISAKV	438934	P1L	HLA-class II	DRB1*∗*0407									

T-cell epitopes in this table are a subset of those provided in Additional File S1.
